# Testing the reproducibility and robustness of the cancer biology literature by robot

**DOI:** 10.1098/rsif.2021.0821

**Published:** 2022-04-06

**Authors:** Katherine Roper, A. Abdel-Rehim, Sonya Hubbard, Martin Carpenter, Andrey Rzhetsky, Larisa Soldatova, Ross D. King

**Affiliations:** ^1^ Manchester Institute of Biology, University of Manchester, Manchester, UK; ^2^ Department of Chemical Engineering and Biotechnology, University of Cambridge, Cambridge, UK; ^3^ Department of Medicine, University of Chicago, Chicago, IL, USA; ^4^ Department of Computing, Goldsmiths University of London, London, UK; ^5^ Department of Computer Science and Engineering, Chalmers University of Technology, Gö‌teborg, Sweden; ^6^ Department of Biology and Biological Engineering, Chalmers University of Technology, Gö‌teborg, Sweden; ^7^ Alan Turing Institute, London NW1 2DB, UK

**Keywords:** testings, reproducibility, robustnesses, cancer, biology, literature

## Abstract

Scientific results should not just be ‘repeatable’ (replicable in the same laboratory under identical conditions), but also ‘reproducible’ (replicable in other laboratories under similar conditions). Results should also, if possible, be ‘robust’ (replicable under a wide range of conditions). The reproducibility and robustness of only a small fraction of published biomedical results has been tested; furthermore, when reproducibility is tested, it is often not found. This situation is termed ‘the reproducibility crisis', and it is one the most important issues facing biomedicine. This crisis would be solved if it were possible to automate reproducibility testing. Here, we describe the semi-automated testing for reproducibility and robustness of simple statements (propositions) about cancer cell biology automatically extracted from the literature. From 12 260 papers, we automatically extracted statements predicted to describe experimental results regarding a change of gene expression in response to drug treatment in breast cancer, from these we selected 74 statements of high biomedical interest. To test the reproducibility of these statements, two different teams used the laboratory automation system Eve and two breast cancer cell lines (MCF7 and MDA-MB-231). Statistically significant evidence for repeatability was found for 43 statements, and significant evidence for reproducibility/robustness in 22 statements. In two cases, the automation made serendipitous discoveries. The reproduced/robust knowledge provides significant insight into cancer. We conclude that semi-automated reproducibility testing is currently achievable, that it could be scaled up to generate a substantive source of reliable knowledge and that automation has the potential to mitigate the reproducibility crisis.

## 1. Introduction

### 1.1. The reproducibility crisis

Ever since the seventeenth-century scientific revolution a fundamental pillar of science has been the requirement for reproducible results [1]. However, despite reproducibility being fundamental to science, the reproducibility of relatively few biomedical results is currently tested; and when reproducibility is tested, difficulty is often experienced in observing reproducibility [2–9]. This situation is termed the ‘reproducibility crisis’: ‘the ability to reproduce experiments is at the heart of science, yet failure to do so is a routine part of research’ [10]; ‘More than 70% of researchers have tried and failed to reproduce another scientist's experiments, and more than half have failed to reproduce their own experiments' [11].

There are a number of reasons for difficulty in reproducing published results: the original result may have been very specific and only true under specific circumstances or the original results may not have been described in sufficient detail to enable reproducibility, stochasticity in the results, etc. Scientific fraud is another possible reason, but this is probably relatively rare [12].

The most direct solution to the reproducibility crisis would be for more scientists to attempt to reproduce other scientists' results. However, there are strong sociological and career disincentives against this: it is hard to get funding for such work, it is hard to publish such studies, authors can react badly to having their results doubted, etc. [13]. Attempts have also been made to identify what factors are important in reproducibility [14,15].

Recognition of the reproducibility crisis has led to multiple initiatives; for example, the Meta-Research Innovation Center at Stanford University (https://metrics.stanford.edu/), the National Research Council of the Netherlands (NOW) Replication Studies pilot programme [16] and the Reproducibility Project Cancer Biology (RPCB) [7–9]. However, these initiatives are limited in extent, and their significance is still to be determined.

Given the high cost and difficulty involved in confirming experimental results, and the current funding model, it is unlikely that human scientists will ever experimentally confirm more than a small fraction of published results. We therefore argue *that the only feasible way to increase the proportion of reproduced results is to automate the process*. To achieve such automation, it will be necessary to integrate text mining (to extract results from the literature) and artificial intelligence-based laboratory automation (to experimentally test the reproducibility of the results).

### 1.2. Forms of experimental confirmation

Here, we recognize distinctions between results that are ‘repeatable’, ‘reproducible’ and ‘robust’. The International Vocabulary of Metrology [17,18] defines ‘repeatability’ as ‘precision in measurements under conditions that include the same measurement procedure, same operators, same measuring system, same operating conditions and same location, and replicate measurements on the same or similar objects over a short period of time’ [19]. ‘Precision’ is defined as ‘closeness of agreement between measured quantities obtained by replicate measurements on the same or similar objects under conditions of repeatability or reproducibility’ [19]. We believe that most published biomedical results are repeatable: laboratories can generally replicate their own published results. Here, we operationally define a statement about cancer from the literature to be ‘repeatable’ if in one set of our semi-automated experiments (same protocol/cell line) we found statistically significant evidence for a result.

‘Reproducibility’ is defined as ‘precision in measurements under conditions that may involve different locations, operators, measuring systems and replicate measurements on the same or similar objects. The different measuring systems may use different measurement procedures’. It is a lack of reproducibility in published biomedical results that is causing the crisis. Here, we operationally define a statement to be ‘reproducible’ if, in our semi-automated experiments, we find the same result as automatically extracted from the literature using our standard experimental approach, and using the same cell line as was originally used.

The term ‘robust’ does not seem to be as formally defined, but results are generally described as ‘robust’ when they are more generally replicable than the above definition of ‘reproducible’ [20,21]. For robust results, the basic biological systems may be different, as well as the experimental apparatus and protocol; however, the conclusions are in agreement. We operationally define a statement about cancer to be ‘robust’ if in our semi-automated experiments we find the same result as automatically extracted from the literature using our standard experimental approach, but using a different cell line from the one that was originally used.

These definitions are consistent with existing formal definitions [17–19]. However, they differ from those proposed in the US National Academies of Sciences/Engineering/Medicine report [6]. This report proposes that ‘*reproducibility* is obtaining consistent results using the same input data; computational steps, methods, and code; and conditions of analysis. This definition is synonymous with "computational reproducibility"’. ‘*Replicability* is obtaining consistent results across studies aimed at answering the same scientific question, each of which has obtained its own data’. We do not follow these definitions as their emphasis is data analysis/computational. The US National Academies of Sciences/Engineering/Medicine definition of replicability is close to what we define as robust reproducibility.

Our operational definitions of ‘repeatable’, ‘reproducible’ and ‘robust’ enable the practical automation of testing for reproducibility, as different textual statements (propositions) can be tested using the same standard experimental protocol and conditions, making the laboratory automation easier to implement. It is currently impossible to automate experiments that closely replicate the whole spectrum of original published experiments because: many published experimental protocols are incomplete, i.e. they do not contain sufficient information to enable even human scientists to reliably repeat them [22,23]—the use of natural language to describe protocols, with all their inherent ambiguities exacerbates this problem [22,23]; even if a published protocol is completely specified for a human scientist, it is currently not possible with existing text-mining technology to extract sufficient information for a robot to implement the protocol in a laboratory; and it is not feasible with existing laboratory automation to more fully test the robust reproducibility of statements by executing a wide variety of orthogonal experimental procedures.

## 2. Results

**Figure 1.**
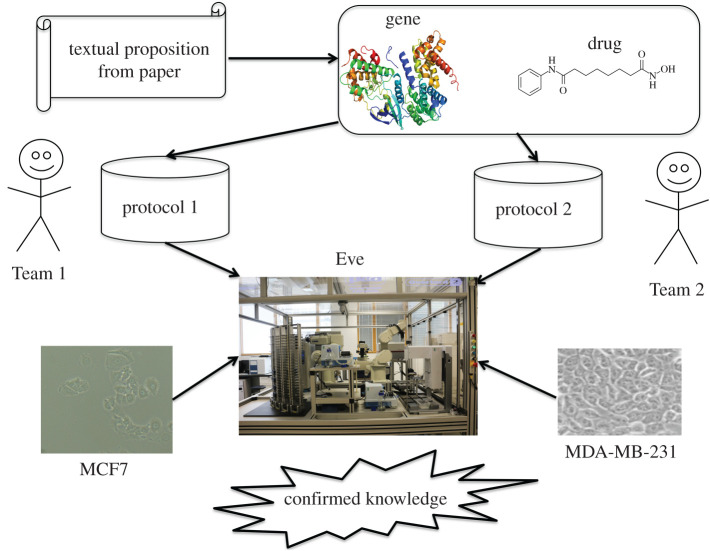
The overall process of testing the reproducibility and robustness of the cancer biology literature by robot. First, text mining is used to extract statements about the effect of drugs on gene expression in breast cancer. Then two different teams semi-automatically tested these statements using two different protocols, and two different cell lines (MCF7 and MDA-MB-231) using the laboratory automation system Eve.

Here, we describe the semi-automated experimental testing of textual statements (propositions) taken from the scientific literature. The overall methodology is shown in figure 1.

### 2.1. Text mining

We focused on textual statements (propositions) taken from the breast cancer literature that were predicted to describe experimental results regarding a change of gene expression in response to a drug (small molecule) treatment. We chose to test such statements because of their medical importance, and because it was expected to be possible to test them using laboratory automation.

A corpus of 12 260 full papers on breast cancer was constructed as part of our work for the Big Mechanism program [24]. To form the corpus, full papers were retrieved from the PubMed Central Open Access repository using ‘breast cancer’ and its synonyms as keywords, combined with names of breast cancer cell lines, e.g. ‘T-47 D’, ‘MCF-7’ (and their variants) [25,26]. These papers were then processed to automatically extract information in a form of ‘index cards’: about 35 925 statements predicted to describe experimental results regarding the change of expression of genes in response to drug treatment. The index cards are ‘json’ files. The content of each card holds information about a statement extracted from the literature. Five main pieces of information are provided: the meta-data, evidence, interaction, submitter and the identifier for the paper the index card is based on.

We used tools from the UK National Centre for Text Mining [25–27]. To extract events, we employed named entity recognition (NER) methods integrated into a unified processing pipeline, which enables the development and execution of reconfigurable, modular NER and event extraction workflows. For event extraction, we applied the machine-learning-based EventMine [27]. EventMine finds trigger words indicating events (e.g. *inhibits*), which are assigned event types (e.g. *negative regulation*). This process is described in [25,26].

### 2.2. Heuristic text filtering

Resource constraints meant that it was not possible to experimentally test all the extracted statements. We therefore chose to select statements of greater biomedical significance. Several heuristics were used to select these (see Material and methods). We first selected events with both qualities ‘entity : simple_chemical’ and ‘event : gene_expression’. This eliminated all events in which gene expression was not affected by a small molecule. The results were further filtered against a list of all genes in two systems biology models of breast cancer: one involving RAS signalling and the other ESR1 signalling. The motivation for this was to examine gene expression in genes known to be important in breast cancer. Finally, statements were filtered against lists of compounds known to be either commercially easily available or unsuitable. This resulted in a set of events of format ‘compound affects gene expression’, known to be in our models, which were possible to test using available compounds. Compounds were manually checked to identify those known to be used or under investigation as cancer therapeutics and those known to be common dietary supplements. The output of filtering was 74 events regarding genes of interest and involving cancer therapeutics or dietary supplements. The full list is presented in table 1.

**Table 1.** The list of statements about the effect of a drug on gene expression levels (textual propositions) tested for reproducibility and robustness.

	gene	drug	id
1	AKT1	4OHT	PMC3711340_E360
2	AKT1	curcumin	PMC4708990_E2037
3	AKT1	EGCG	PMC2927993_E10333
4	ATF4	NAC	PMC4546701_E754
5	BIRC5	curcumin	PMC2756684_E6964
6	BIRC5	daidzein	PMC2944964_E8929
7	BIRC5	doxorubicin	PMC2649216_E5319
8	BIRC5	paclitaxel	PMC2826345_E10033
9	BRCA2	daidzein	PMC2361140_E3414
10	BRCA1	indol-3-carbinol	PMC4346871_E712
11	CASP3	quercetin	PMC2712839_E6241
12	CCND1	4OHT	PMC2882356_E7162
13	CCND1	curcumin	PMC3206621_E15380
14	CCND1	resveratrol	PMC4000631_E146
15	CCND1	SAHA	PMC3355273_E18930
16	CCND1	salinomycin	PMC4631341_E1017
17	CTNNB1	cordycepin	PMC3784440_E402
18	CTNNB1	curcumin	PMC3706856_E361
19	CTNNB1	EGCG	PMC2933702_E10181
29	EGFR	curcumin	PMC3206621_E15401
21	EGFR	doxorubicin	PMC3181057_E14848
22	ERBB2	curcumin	PMC4003153_E459
23	ERBB3	fulvestrant	PMC2875575_E10985
24	ESR1	4OHT	PMC2882356_E7158
25	ESR1	curcumin	PMC2705850_E4569
26	ESR1	EGCG	PMC2967543_E11055
27	ESR1	fulvestrant	PMC3139592_E14864
28	ESR1	pterostilbene	PMC4134202_E1283
29	ESR1	quercetin	PMC4228827_E129
30	ESR1	resveratrol	PMC3521661_E722
31	HDAC1	curcumin	PMC3625766_E1801
32	HDAC1	resveratrol	PMC3625766_E1802
33	HDAC1	SAHA	PMC3498753_E565
34	HIF1A	doxorubicin	PMC4024011_E700
35	HIF1A	melatonin	PMC4123875_E984
36	HIF1A	zoledronic_acid	PMC4496173_E126
37	HSP90	quercetin	PMC3652296_E1279
38	IL8	NAC	PMC4463759_E1355
39	MAPT	4OHT	PMC2917038_E8406
40	MAPT	fulvestrant	PMC2917038_E8306
41	MELK	paclitaxel	PMC3857210_E1352
42	MMP-2	silibinin	PMC4006687_E357
43	MMP-9	curcumin	PMC4176907_E1376
44	MMP-9	silibinin	PMC4196436_E1516
45	MTOR	SAHA	PMC3840459_E1427
46	NFK1B	quercetin	PMC3747514_E565
47	p21	doxorubicin	PMC3765348_E744
48	p21	paclitaxel	PMC2394338_E3767
49	p21	resveratrol	PMC2364738_E2929
50	p21	vinorelbine	PMC2394338_E3826
51	p27	curcumin	PMC3706856_E382
52	p300	curcumin	PMC3255482_E16909
53	p53	caffeic_acid	PMC2928446_E12078
54	p53	doxorubicin	PMC4228062_E94
55	p53	etoposide	PMC4400643_E1283
56	p53	hesperidin	PMC4177652_E1404
57	p53	resveratrol	PMC2928446_E12079
58	PDK1	curcumin	PMC4192446_E1344
59	PGR	letrozole	PMC1064088_E125
60	PTEN	resveratrol	PMC2957324_E13190
61	PTEN	silibinin	PMC3148510_E16237
62	RASSF1	4OHT	PMC3977804_E166
63	STAT3	curcumin	PMC3584822_E1221
64	STAT3	doxorubicin	PMC4589559_E1201
65	STAT3	paclitaxel	PMC4467444_E173
66	STK11	honokiol	PMC3496153_E906
67	TNF	paclitaxel	PMC2830051_E9591
68	TXNIP	resveratrol	PMC3733924_E363
69	uPA	EGCG	PMC4006687_E360
70	uPA	silibinin	PMC4006687_E360
71	VEGFA	EGCG	PMC3708553_E323
72	VEGFA	melatonin	PMC3708553_E323
73	VEGFA	NAC	PMC3929894_E1687
74	VEGFA	paclitaxel	PMC3682088_E5

### 2.3. Repeatable changes in gene expressions

Using the artificial intelligence (AI)-based laboratory automation system ‘Eve’ [28], we experimentally tested the 74 statements obtained from heuristic text filtering. We used two breast cancer cell lines, MCF7 and MDA-MB-231 (ATCC, USA). MCF7 is the most studied human breast cancer cell line, with over 25 000 scientific publications using it [29]. It originated as an invasive breast ductal carcinoma, is ‘luminal’ type and oestrogen receptors (ERs) are present. The breast cancer cell line MDA-MB-231 is ‘basal’ type and triple negative, i.e. missing three markers: ER, progesterone receptor (PR) and HER2/Neu oncogene [30].

Eve was originally designed as a ‘robot scientist’, an AI-directed laboratory automation system that automatically: originates hypotheses to explain observations, devises experiments to test these hypotheses, physically runs the experiments using laboratory robotics, interprets the results to change the probability of hypotheses and then repeats the cycle [28,31]. Eve has multiple functionalities and was originally designed for automatic early-stage drug development. In this work, we did not run Eve in a closed-loop automation manner. This was because the hypotheses to test were taken directly from the literature, and there was no need to modify them based on experimental results.

Using Eve we applied two different but closely related protocols. Each protocol was run by a different team (team 1 and team 2) using multiple replicates. The same laboratory was used by both teams, but the teams worked months apart. The protocols were based on real-time polymerase chain reaction (rtPCR) to measure the expression of a targeted gene. The protocols were designed for moderate throughput semi-automated experimentation. For full details, see Material and methods.

The results for each statement were first evaluated for repeatability, i.e. could we repeatedly obtain this same result using Eve? Specifically, we defined a statement to be repeatable if with Eve one team in one cell line found statistically significant evidence for replication of a result. To decide on significance, we applied a classical sign test: the number of replicates with increased expression versus the number of replicates with decreased expression. This test is straightforward and robust to assumptions about the underlying distribution. We found that 43 statements had significant evidence for repeatability (at least one of the teams on one of the cell lines; *p* < 0.05). These are shown in table 2.

**Table 2.** The list of repeatable results. These drugs were found to produce statistically significant changes in the expression of the genes. Human reading—what was found by human annotators: text—is the direction of change of gene expression (↑ increase, stimulation; ↓ decrease, inhibition); MCF7—whether the change was found using the MCF7 cell line; MDA—whether the change was found using the MDA-MB-231 cell line. Text mining—the direction of change of gene expression identified automatically by the computer. MCF7—the results of the robotic experiments using the MCF7 cell line. MDA—the results of the robotic experiments using the MDA-MB-231 cell line. Team 1—the statistical significance found by team 1; team 2—the statistical significance found by team 2; sign—the direction of change of gene expression.

	gene	drug	human reading			text mining	MCF7				MDA			
			text	MCF7	MDA	sign	team 1	sign	team 2	sign	team 1	sign	team 2	sign
1	AKT1	4OHT	↓^a^	Y	N	—	—	—	0.0009766	↓	—	—	—	—
2	BIRC5	doxorubicin	↑	N	N	↑	—	—	0.0004883	↓	—	—	—	—
3	BRCA2	daidzein	—^b^	Y	N	↑	—	—	—	—	—	—	0.0175781	↑
4	BRCA1	indol-3-carbinol	↑	Y	N	↑	—	—	—	—	—	—	0.0009766	↓
5	CASP3	quercetin	↑	N	N	↑	—	—	—	—	—	—	0.0004883	↑
6	CCND1	curcumin	↓	Y	Y	↓	—	—	—	—	—	—	0.0039063	↑
7	CCND1	SAHA	↓	Y	N	—	0.0039063	↓	0.0002441	↓	0.0019531	↓	0.0268555	↓
8	CTNNB1	cordycepin	↓^c^	Y	N	—/↓	—	—	—	—	0.03125	↓	—	—
9	CTNNB1	curcumin	↓	Y	Y	↓	—	—	0.0097656	↓	—	—	—	—
10	CTNNB1	EGCG	↓	N	N	↓	—	—	—	—	0.03125	↓	—	—
11	EGFR	curcumin	↓	N	N	↓	—	—	—	—	—	—	0.0175781	↑
12	EGFR	doxorubicin	↓	N	N	↓	—	—	—	—	—	—	0.0002441	↓
13	ERBB3	fulvestrant	↑	Y	N	↓	—	—	0.0004883	↑	—	—	—	—
14	ESR1	4OHT	↓	Y	N^d^	↓	—	—	0.03125	↓	—	—	—	—
15	ESR1	pterostilbene	↓	Y^e^	N	↓	—	—	0.0009766	↓	—	—	—	—
16	ESR1	quercetin	↓	N	N	↓	—	—	0.015625	↓	—	—	—	—
17	HIF1A	doxorubicin	↓	N	N	↓	—	—	0.0002441	↓	—	—	—	—
18	MAPT	4OHT	↑	Y	N	↑	—	—	—	—	0.03125	↑	—	—
19	MAPT	fulvestrant	↓	Y	N	↓	—	—	0.0029297	↓	—	—	—	
20	MMP-2	silibinin	↓	N	N	↓	—	—	—	—	—	—	0.0175781	↓
21	MMP-9	curcumin	↓^f^	N	N	↓	—	—	0.0078125	↓	—	—	0.0703125	↓
22	MTOR	SAHA	↓	unclear	unclear	↓	—	—	—	—	—	—	0.0009766	↑
23	NFK1B	quercetin	↓	N	N	↓	—	—	0.0019531	↓	—	—	0.0439453	↑
24	p21	doxorubicin	↑	N	N	↑	—	—	0.015625	↓	—	—	—	—
25	p21	paclitaxel	↑	N	N	↑	0.015625	↑	—	—	—	—	—	—
26	p21	resveratrol	↑	Y	N	↑	—	—	—	—	0.015625	↓	—	—
27	p300	curcumin	↓	N	N	↓	—	—	—	—	—	—	0.03125	↑
28	p53	caffeic acid	↑	N	N	↑	—	—	—	—	—	—	0.0439453	↑
29	p53	etoposide	↑^g^	Y	Y	↑	0.03125	↓	0.03125	↓	0.03125	↓	0.0053711	↓
30	p53	hesperidin	↑^h^	N	N	↑	—	—	0.0039063	↓	0.0703125	↓	—	—
31	p53	resveratrol	—	Y	N	↑	—	—	—	—	0.0175781	↓	—	—
32	PDK1	curcumin	↓^a^	N	N	↓	—	—	0.0039063	↓	—	—	—	—
33	PGR	letrozole	↓	N	N	↓	—	—	0.0010376	↓	—	—	—	—
34	PTEN	resveratrol	↑	Y	N	↑	—	—	6.87 × 10^−5^	↓	—	—	0.0019531	↓
35	PTEN	silibinin	—	N	N	↓	—	—	3.05 × 10^−5^	↓	—	—	—	—
36	STAT3	curcumin	—	Y	Y	↓	—	—	—	—	—	—	0.03125	↑
37	STAT3	doxorubicin	↑	Y	N	↑	—	—	—	—	—	—	0.0039063	↑
38	STAT3	paclitaxel	↓^i^	N	N	—	0.015625	↑	—	—	—	—	—	—
39	TXNIP	resveratrol	↑↓^j^	Y	N	↑	—	—	0.0004883	↓	—	—	—	—
40	uPA	EGCG	↓	N	N	↓	—	—	—	—	—	—	0.0009766	↑
41	uPA	silibinin	—	N	N	↓	—	—	0.0001221	↓	—	—	—	—
42	VEGFA	melatonin	↓	N	N	↓	—	—	—	—	—	—	0.0004883	↓
43	VEGFA	NAC	—	N	N	↓	—	—	—	—	—	—	0.0078125	↓

^a^Inhibition in paper ‘not significant’.

^b^Refers to a different paper.

^c^No effect claimed in text, but appears in a figure.

^d^Gene missing.

^e^MCF7 with constructs.

^f^The paper is a review.

^g^The paper does not describe TP53, but rather a splice variant of TP53.

^h^Refers to another paper with NALM-6 cells.

^i^phospho Stat3.

^j^Biphasic depending on concentration.

### 2.4. Manual testing of the text mining

To evaluate the automated text mining that found the 43 repeatable statements, we manually read the original papers from which the statements were extracted. We assessed whether the compound was reported to cause inhibition or an increase in expression of the gene, and whether this statement was about MCF7 or MDA-MB-231 cells (table 2). We found that the text-mining software had generally done a good job in identifying statements that describe experimental results regarding the change of gene expression in response to drug (small molecule) treatment. Only four statements were false positives—where the human readers failed to identify the proposition recognized by the text mining (table 2). The text-mining software also performed well in identifying the correct direction of change in the propositions. In only one case, for the drug fulvestrant and the gene ERBB3, was the sign extracted wrongly.

The text-mining software did not attempt to determine cell culture type referred to in the statements beyond looking for the keywords ‘MCF7’ and ‘MDA-MB-231’ in papers. Automatically determining cell culture type is difficult as this experimental detail is often far from the textual statement about the effect of a drug. Manual reading found that 19 statements referred to experiments using MCF7. In four cases, the statements referred to experiments using MDA-MB-231.

### 2.5. Experimental testing of reproducibility and robustness

We operationally defined a statement about cancer to be ‘reproducible’ if, using Eve, we found statistically significant evidence for a change of gene expression in the same cell line, and in the same direction as in the original paper. As with replicability, we used a sign test to decide significance. We found statistically significant evidence for experimental reproducibility of six statements (table 3).

**Table 3.** The list of reproducible results. These effects of drugs on gene expression levels were successfully read from the literature by text mining and were experimentally confirmed using semi-automatic robotic experiments.

cell	↑↓	drug	gene/protein	significance
***MCF7***	**↓**	***4OHT*** is a selective oestrogen receptor modulator (SERM) of the triphenylethylene group and the major active metabolite of the breast cancer drug tamoxifen.	***ESR1*** is the gene product of oestrogen receptor 1, a nuclear receptor activated by the sex hormone oestrogen.	It is of clinical interest that 4OHT both inhibits the receptor and inhibits the expression of ESR1. It is unclear if this effect is beneficial in cancer treatment or not. ESR1 is missing from MDA-MB-231.
***MCF7***	**↓**	***4OHT***	***AKT1*** is a serine/threonine-specific protein kinase that regulates cellular survival and metabolism. AKT is associated with tumour cell survival, proliferation and invasiveness.	In cancer treatment it is generally considered desirable to inhibit AKT.
***MCF7***	**↓**	***SAHA*** (suberoyl+anilide + hydroxamic acid; vorinostat) is a histone deacetylase (HDAC) inhibitor. The molecular mechanisms underlying the response to HDAC inhibitors in cancer patients is not fully understood [32].	***CCND1* (cyclin D1)** is the gene product involved in the regulation of cyclin-dependent (CDK) kinases. Mutations in CCND1, or alterations in its expression, have been shown to have a role in tumorigenesis. There is evidence that CCND11 regulates the tumour suppressor protein Rb, making it a target for the development of anti-cancer treatments.	This statement has perhaps the strongest evidence for reproducibility (table 3): it was reproduced by both teams and robustly reproduced by both groups. The robust reproducibility of this result may point to the mechanism of action of SAHA against cancer as acting through CCND1 and RB.
***MCF7***	**↓**	***Curcumin*** is a polyphenolic compound derived from the Indian spice turmeric plant. Its pharmacological properties are complex and controversial.	***CTNNB1***—the gene product, β-catenin, is involved in regulation and coordination of cell–cell adhesion and gene transcription. Mutations and overexpression of CTNNB1 are associated with many cancers.	In cancer treatment it is generally considered desirable to inhibit CTNNB1, so the inhibition of CTNNB1 is a desirable effect of curcumin.
***MCF7***	**↑**	***Fulvestrant*** is a selective oestrogen receptor degrader. It is used to treat hormone receptor-positive metastatic breast cancer.	***ERBB3***—the gene product is a member of the epidermal growth factor receptor (EGFR/ERBB) family of receptor tyrosine kinases. Changes in its expression are associated with targeted therapeutic resistance in numerous cancers.	We did not observe changes in expression in MDA-MB-231, which is consistent with action through the oestrogen receptor. The reproduced observation of increased ERBB3 expression with fulvestrant may be of concern in cancer treatment.
***MCF7***	**↓**	***Fulvestrant***	***MAPT***—the primary role of the gene product, microtubule-associated protein tau, is in maintaining the stability of microtubules in axons. Pathologies and dementias of the nervous system, such as Alzheimer's disease, are associated with MAPT.	The inhibitory effect of fulvestrant on MAPT may cause unwanted neural side-effects.

We investigated two forms of robust reproducibility: minor and major. In minor robustness, the proposition in the paper was found using MCF7, yet was confirmed by Eve in MDA-MB-231. There were four cases of minor robustness (table 4). In major robustness, the original proposition was about neither MCF7 nor MDA-MB-231, but we found conformational evidence in either MCF7 or MDA-MB-231 cells. There were 12 cases of major robustness (table 5).

**Table 4.** The list of minor robust results. These statements about the effect of drugs on gene expression were about MCF7 cells but were confirmed in MDA-MB-231 cells [33,34].

↑↓	drug	gene	notes
**↓**	***SAHA***	***CCND***	Interestingly, this is the only case where the result was also confirmed in MCF7, i.e. it was reproduced and robustly confirmed. It is unclear why in the other cases, where the original paper reported an effect in MCF7, we only saw an effect in MDA-MB-231.
**↓**	***cordycepin***	***CTNNB1***	Cordycepin is a derivative of the nucleoside adenosine. Our interpretation of the evidence in [35], where the statement arose, is that CTNNB1 (protein) expression level is reduced [35, fig. 2].
**↑**	***4OHT***	***MAPT***	This statement is interesting as the increased expression of the gene product of MAPT by 4OHT may cause unwanted side-effects in cancer treatment
**↑**	***doxorubicin***	***STAT3***	Doxorubicin (DXR) is an anti-cancer drug, a 14-hydroxylated version of daunorubicin. Doxorubicin interacts with DNA by intercalation and inhibition of macromolecular biosynthesis. STAT3 is a transcription factor which plays a key role in many cellular processes such as cell growth and apoptosis. STAT3 may promote oncogenesis by being constitutively active.

**Table 5.** The list of major robust results. In major robustness the original textual statement was about neither MCF7 nor MDA-MB-231 cells. Notes: In one case, ↑PTEN/resveratrol, we see a consistently opposite effect in both MCF7 and MDA-MB-231 cells to that observed in the paper in MCF7 cells. This observation does not invalidate the replicability of the original result, but it does raise questions about its reproducibility. PTEN (phosphatase and tensin homologue) acts as a tumour suppressor gene. Up to 70% of primary prostate tumours lose one *PTEN* allele and retain the other copy [36]. Resveratrol (3,5,4′-trihydroxy-trans-stilbene) is a stilbenoid, a natural plant product. Resveratrol is associated with possible life longevity. The inhibition of PTEN by resveratrol is potentially of clinical concern.

↑↓	drug	gene	notes
**↑**	***quercetin***	***CASP3***	The gene product of CASP3 protein is a cysteine–aspartic acid protease (caspase). Activation of caspases plays a central role in the execution phase of cell apoptosis. Quercetin is a plant flavonol; quercetin supplements have been promoted for the treatment of cancer.
**↓**	***EGCG***	***CTNNB1***	EGCG (epigallocatechin gallate) is the most abundant catechin in tea.
**↓**	***doxorubicin***	***EGFR***	The gene product of EGFR (epidermal growth factor receptor) is a receptor for members of the epidermal growth factor family (EGF family). Mutations that lead to EGFR overexpression are associated with a number of cancers.
**↓**	***quercetin***	***ESR1***	—
**↓**	***doxorubicin***	***HIF1A***	HIF1A is a subunit of a heterodimeric of hypoxia-inducible factor 1, a transcription factor that responds to decreases in available oxygen in the cellular environment, or hypoxia. (The 2019 Nobel Prize in Physiology or Medicine was partly awarded for discovery of this function.) The dysregulation and overexpression of *HIF1A* have been implicated in cancer.
**↓**	***silibinin***	***MMP-2***	The gene product of MMP-2 is a zinc metalloproteinase (matrix metalloproteinase-9). It cleaves collagen type IV. Degradation of collagen IV in basement membrane and extracellular matrix facilitates tumour progression, including invasion, metastasis, growth and angiogenesis.
**↓**	***curcumin***	***MMP-9***	The gene product of MMP-9 is a zinc metalloproteinase that cleaves gelatin types I and V and collagen types IV and V.
**↑**	***paclitaxel***	***p21***	Paclitaxel is a natural plant product used to treat many cancers. Its mode of action is through targeting tubulin. Paclitaxel stabilizes the microtubule polymer and protects it from disassembly; chromosomes therefore fail to achieve a metaphase spindle configuration.
**↑**	***caffeic acid***	***P53***	Caffeic acid is a natural plant product that is being investigated for anti-cancer treatment. The observation of significantly increased promotion of P53 in MDA-MB-231 may be linked to the fact that this gene is mutated and expressed at high levels relative to MCF7 cells.
**↓**	***curcumin***	***PDK1***	The gene product of PDK1 (protein 3-phosphoinositide-dependent protein kinase-1). It is a central kinase in cell signalling.
**↓**	***letrozole***	***PGR***	The gene product of PGR is a progesterone receptor. Mutations in PGR are associated with breast cancer. Letrozole is an aromatase inhibitor that is used in the treatment of hormonally responsive breast cancer. Our observation of inhibition in MCF7, but not MDA-MB-231 (table 2), is consistent with MDA-MB-231 lacking ESR.
**↓**	***melatonin***	***VEGFA***	VEGFA (vascular endothelial growth factor A) is in the platelet-derived growth factor family of cystine-knot growth factors. The VEGF family stimulate cellular responses by binding to tyrosine kinase receptors. Melatonin (*N*-acetyl-5-methoxy tryptamine) is a hormone involved in the human sleep–wake cycle. It is a commonly used sleep aid. Our result robustly reproduces the evidence for repurposing the known safe drug melatonin.

### 2.6. Novel knowledge about changes in gene expressions

In two cases, we found statistically significant results where the original paper stated that the result was not statistically significant: *drug 4OHT inhibits gene AKT1 expression in cell line MCF7* and *drug curcumin inhibits gene PDK1 expression*. The first case is one of reproducibility in MCF7, the second of robust reproducibility. Eve has therefore semi-automatically provided the first statistically significant evidence for these medically important statements.

It is often stated that because machines do not make mistakes they cannot therefore make serendipitous discoveries. This argument is incorrect as machines do make mistakes. In two cases, the text-mining software incorrectly identified statements that it believed to involve the drug silibinin inhibiting genes: *drug silibinin inhibits gene PTEN expression* and *drug silibinin inhibits gene* *uPA* *expression.* Although these statements were not found by human readers in the original papers, statistically significant experimental evidence was found for these statements in the cell line MCF7, i.e. they were both repeatable.

### 2.7. Limitations

Our approach is limited in a number of ways:

— The hardware and software limitations of Eve mean that the experiments were only semi-automated, not fully automated.
— The text mining is only capable of extracting simple information from texts.
— We only tested simple propositions of the form compound X affects the expression of gene Y. The results of scientific papers contain much more sophisticated and nuanced results. Such results are currently difficult to analyse using text mining/AI; it is also more difficult to automate the replication of such results.
— The experiments were restricted to two related PCR protocols.
— We only investigated two cell lines in one form of cancer—breast cancer.

## 3. Discussion

The cancer literature is both vast and sparse. Tens of thousands of papers have been published on cancer cell biology, yet, because of the underlying complexity of the biology and the systemic disincentives to replication in science, very little of the literature reports the direct replication of results from other papers. When different laboratories attempt to reproduce others' work, it is often in different cell lines, different populations or using different techniques. This makes it very difficult to know how relevant a statement in the literature that used system X and protocol P is to system Y and what to expect with protocol Q. The cancer literature is also sparse: owing to high heterogeneity, countless different experimental systems are used. A further complication is genetic evolution of and heterogeneity within cell lines [37], which means that our MCF7 and MDA-MB-231 cell lines may differ significantly from the same denoted cell lines in papers.

We have demonstrated the semi-automated testing of literature statements for reproducibility and robustness. In the cases where we found reproducibility or robustness, the results confirm the original literature statements and provide evidence for their correctness. However, for the cases where we failed to find reproducibility or robustness, this does prove that statements are not reproducible or robust. There are many reasons for the failure to reproduce results that are replicable in another laboratory. These may include: the original biological system was slightly different, e.g. cell lines are known to alter in different laboratories under different conditions; the original protocol was slightly different from the one that we used; our experimental results are incorrect; and our assumed monotonicity between gene and protein expression (so an observed increase in protein level, say by western blot, is evidence for an increased level of gene expression).

We argue that a key step towards reducing the sparseness, heterogeneity and lack of reproducible results is for the general automated testing of statements from the cancer literature in model cancer cell lines, which would generate a source of reproducible/robust knowledge about cancer biology. Many papers are based on results produced in cancer cell lines. If these results could be confirmed by other semi-automated laboratories, these results could then be confirmed in tissues acquired from patients, and eventually in patients themselves. The advantages of this approach are as follows.

— Automation side-steps the sociological and career disincentives for replication.
— Automation is cheaper and faster than manual replication as robots can work longer and faster than human scientists. Automation also enables miniaturization of experiments where humans would be likely to make mistakes. For example, pipetting 384-plate quantitative PCR (qPCR) experiments with complex layouts.
— Automation makes experimental replication technically easier, as laboratory robotics are more accurate at executing experiments than humans; they also record experiments in much greater semantic detail [31].
— The use of standard cancer lines and protocols controls for the heterogeneity of the results and ensures that experimental results are comparable on the same biological systems.
— The use of standard cancer lines and protocols controls also enables experimental results from different biological systems to be integrated together in a single biological system.
— Such systems would enable a large body of reproducible and robust experimental results to be accumulated about specific cancers and potentially cancer as a whole.
— Automation aids in following the FAIR (findability, accessibility, interoperability and reusability) principles for publishing data [38].

In this paper, we tested 74 (0.2%) of the 35 925 statements identified using text mining in a period of approximately 18 months. We argue that through the use of greater laboratory automation it would be eminently feasible to test the remaining 35 846 statements, as this would involve only straightforward up-scale engineering. We estimate that this could be done in 5 years at a cost of approximately US$10 M. Such a study would cost US$278 per statement tested for reproducibility. This cost is in line with current laboratory automation experimental costs. The main costs would be technical support and laboratory consumables, especially sourcing the test compounds. For this cost, it would also be possible to have human experts to sanity check the statements to be robotically tested for reproducibility. The recently published RPCB study [7–9] cost US$52 574 per completed paper. However, this was the cost of manually reproducing the main results in the papers and includes the cost of corresponding with the original authors. In the proposed fully automated study, it would be possible to automatically contact the original authors to inform them of the conclusion of the reproducibility study, but it would be an interesting text-mining/AI project to automate any more correspondence. The output of such high-throughput statement reproducibility testing would create a unique resource of machine-curated knowledge, which would be a first step towards fully automating the testing of the cell cancer literature for reproducibility and robustness.

To fully achieve the vision of automated literature testing will require technical advances in laboratory robotics, in text mining and in AI. The flexible automated testing of literature results will require the application of adaptable laboratory automation systems capable of executing the same range of experiments that a typical cell biologist can execute. This is technically feasible, as it is now possible to fully automate almost any experimental method that can be manually executed. The best documented such systems are termed ‘cloud automation’ (Strateos, Emerald Cloud, etc.). The use of such automation has the potential to improve the reproducibility of science, as they enable the description of experiments in greater detail and semantic clarity. In such automated laboratories, protocols can be fully formalized and shared—like computer code [23,31]. Advances are also required in text mining, where it will be necessary to extract and semantically tag many more, if not all, of the essential technical and experimental details of papers. This is not possible with current text-mining methods, but with continuing advances in natural language understanding technology, and thanks to the restricted scope of scientific papers and their stereotypical structure, it is reasonable to expect rapid progress in this area.

Finally, the hardest part of fully automating the testing of the cancer cell biology literature will be developing an AI system that understands enough about cell biology both to intelligently interpret the literature and to intelligently design experiments that seek to reproduce the published results, and ultimately to test their general validity. Such a system would end the cancer reproducibility crisis.

## 4. Material and methods

### 4.1. Materials

The cell lines used were MCF7 (Sigma, 86012803) and MDA-MB-231 (ATCC, HTB-26). Compounds were individually ordered (Selleck, Tocris and Sigma). CellsDirect Resuspension and Lysis Buffers (ThermoFisher, 11739010). CCK-8 cell counting kit (Sigma, 96992). NEB Luna Universal Probe One-Step RT-qPCR Kit (NEB, E3006 L). Thermo Fisher single-tube Taqman gene expression assays: AKT1, Hs00178289_m1; ATF4, Hs00909569_g1; BIRC5, Hs00977611_g1; BRCA1, Hs01556193_m1; BRCA2, Hs00609073_m1; CASP3, Hs00234387_m1; CCND1, Hs00765553_m1; CTNNB1, Hs00355049_m1; EGFR, Hs01076092_m1; ERBB2, Hs01001580_m1; ERBB3, Hs00176538_m1; ERBB3, Hs00951444_m1; ERBB3, Hs00951455_m1; ESR1, Hs01046812_m1; ESR1, Hs01046816_m1; HDAC1, Hs00606262_g1; HIF1A, Hs00153153_m1; HSP90, Hs00743767_sH; IL-8, Hs00174103_m1; MAPT, Hs00902194_m1; MELK, Hs01106440_m1; MMP-2, Hs01548727_m1; MMP-9, Hs00234579_m1; MTOR, Hs00234508_m1; NF-KB1, Hs00765730_m1; p21, Hs00355782_m1; p27, Hs01597588_m1; p300, Hs00914223_m1; p53, Hs01034249_m1; PDK1, Hs01561850_m1; PGR, Hs00172183_m1; PTEN, Hs02621230_s1; RASSF1, Hs00176538_m1; STAT3, Hs00374280_m1; STK11, Hs00975988_m1; TNF, Hs01113624_g1; TXNIP, Hs00197750_m1; uPA, Hs01547054_m1; VEGFA, Hs00900055_m1. All code used is available on request.

### 4.2. Team 1 assay methods

#### 4.2.1. Compound treatment and lysate harvesting

A Labcyte Echo 550 was used to plate out each compound on four wells on 384-well cell culture-coated plates (Greiner) to a final concentration of 10 µM. Multidrop liquid dispensers were used to add 25 µl of cells (approx. 3600/µl) to wells. After 24 h growth an Agilent Bravo and Multidrop dispensers were used to aspirate culture medium from three of the four wells and wash cells in Dulbecco's phosphate-buffered saline (dPBS), before lysing with Cells Direct lysis buffer. Lysed cells were pooled and transferred to a 384-well rtPCR plate (Roche) and stored at −80°C until needed. Upon removal from storage lysed cells were immediately heated at to 72°C for 10 min to denature the contents of the lysis buffer.

Culture medium in the remaining well from each quadrant was instead supplemented with a CCK-8 cell counting kit (Sigma) and a BMG Polarstar platereader was used to measure optical density at 450 nM. This measurement was used to track compound lethality.

#### 4.2.2. rtPCR set-up

Cell lysates were diluted 1 : 2 in nuclease-free water. An Agilent Bravo was used to add 3 µl of lysate to individual wells of an uncoated 384-well plate (Corning). An NEB Luna Universal Probe One-Step RT-qPCR kit was used to carry out qPCR reactions. A single master mix containing enzyme mix, buffer and nuclease free water was split into aliquots on a round-bottomed 96-well plate (Nunclon), to which Taqman Gene Expression Assays (ThermoFisher) were manually added. The Bravo was then used to transfer 23.5 µl of reaction mix to the wells with lysate and to mix the lysates and reaction mix, and then transfer 4.5 µl from each mini-master mix to four wells in a 384-well rtPCR plate. Reactions were carried out on a Roche Lightcycler 480 with conditions as follows: one cycle at 55°C for 10 min and 95°C for 1 min, followed by 50 cycles at 95°C for 10 s and 60°C for 30 s.

#### 4.2.3. rtPCR analysis

The comparative CT method (ΔΔCT) uses a reference sample and an endogenous control to determine the relative quantity of target nucleic acid sequence in a sample. ΔΔCT was used to analyse rtPCR results, with GusB used as a control gene. Data were pooled over three repeat experiments, with a sign test used to determine the directionality of changes in expression and a two-tailed *t*-test was used to determine significance of the change.

### 4.3. Team 2 assay methods

#### 4.3.1. Compound treatment and lysate harvesting

A Labcyte Echo 550 was used to plate out 10 μM of each compound onto four wells of a 384-well cell culture-coated plate (Greiner). Multidrop liquid dispensers were used to add 25 µl of cells (approx. 3600 cells µl^−1^) to the wells. Cells were incubated with compounds at 37°C, 5% CO_2_ for 24 h. Culture medium was aspirated from three of the four wells, and the cells were washed in dPBS three times. Agilent Bravo and Multidrop dispensers were used for this. Cells were then lysed with CellsDirect lysis buffer (Invitrogen). Lysed cells were pooled and transferred to a 384-well rtPCR plate (Roche) and stored at −80°C until needed. Upon removal from storage lysed cells were immediately heated to 72°C for 10 min to inactivate the lysis buffer. Lysate was then used for rtPCR.

Culture medium in the remaining wells from each quadrant was supplemented with a CCK-8 cell counting kit (Sigma) and a BMG Polarstar plate reader was used to measure the optical density of the well at 450 nM. This measurement was used to track compound lethality.

#### 4.3.2. rtPCR set-up

Cell lysates were diluted 1 : 2 in nuclease-free water. An Agilent Bravo was used to add 3 µl of lysate to individual wells of an uncoated 384-well plate (Corning). The exact amount of starting RNA was not calculated as we were not comparing between test and control experiments. qPCR reactions were performed using an NEB Luna Universal Probe One-Step RT-qPCR kit, following the manufacturer's directions. A single master mix containing enzyme mix, buffer and nuclease free water was split into aliquots on a round-bottomed 96-well plate (Nunclon). Taqman Gene Expression Assays (ThermoFisher) were manually added. A 23.5 µl aliquot of the reaction mix was transferred to the lysate using the Agilent Bravo and mixed. From each well, 4.5 µl was transferred to four wells in a 384-well rtPCR plate. Reactions were carried out on a Roche Lightcycler 480 with conditions as follows: one reverse transcriptase incubation at 55°C for 10 min and 95°C for 1 min, followed by 50 cycles at 95°C for 10 s and 60°C for 30 s.

#### 4.3.3. Quantitative polymerase chain reaction analysis

The comparative CT method was used to analyse the qPCR results, with GusB used as a control gene. Data were pooled over three repeat experiments, and a sign test used to determine the directionality of changes in expression and a two-tailed *t*-test used to determine significance of the change.

### 4.4. Event filtering

The output of the text-mining process was 35 925 ‘events’. Every event is of the form: a small chemical affecting a gene/protein. Several heuristics were used to filter the events for biological significance.

There are three broad stages to the pipeline. The first stage refined the text mining to more certain statements (table 6). The second stage focused on statements relevant to research interests of the group, on change of gene expression (or protein), and where the compounds where commercially available (table 7). The final manual stage integrated the heuristics, focused on breast cancer and chose cheaper compounds (table 6).

**Table 6.** Stage 1. Every event is of the desired form simple chemical affecting a gene/protein—thus allowing for convenient experimentation. There are no ‘duplicated’ events in the results. Groundings into UniProt and Chebi are attempted, and provided where there is reasonable confidence in their accuracy.

heuristic	matching statements
chemical as object	8084
protein as subject	33 202
grounded proteins as subject	13 219
grounded chemicals as object	6209
chemical object, protein subject	7174
grounded chemical, protein subject	5501
grounded protein and chemical	1999
cell line data, anything allowed—sentence + methods section	5129
cell line data, only ‘known’ names allowed	2363

**Table 7.** Stage 2. Subject protein present as a node in the Petri net model—this checks if the subject protein is present as a node in the Petri net model or not. Subject protein present as a node in the Chicago model—this checks if the subject protein is present as a node in the Boolean model from Chicago or not. Event of type gene expression. Object chemical is known to be commercially available—this is a check that the chemical in question can be purchased at a reasonably plausible price/time scale—conceptually this could be done automatically.

heuristic	matching statements
protein names matching to Petri net model	5340
grounded proteins matching to Petri net model	395
protein names matching to Chicago model	6531
grounded proteins matching to Chicago model	3404
names matching Chicago or Petri net model	9413
grounding match Chicago or Petri net model	3474
gene expression event	9393
known commercially available chemical	2172
known commercially available chemical and Chebi grounded	1911

## Data Availability

All data are available on request. They can be freely copied and reused.
